# Extracellular Vesicles in Diagnosis and Treatment of Pancreatic Cancer: Current State and Future Perspectives

**DOI:** 10.3390/cancers12061530

**Published:** 2020-06-10

**Authors:** J. Spencer Lane, Daniel Von Hoff, Derek Cridebring, Ajay Goel

**Affiliations:** 1 Internal Medicine-Pediatrics, University of Texas Houston, Houston, TX 77030, USA; John.S.Lane@uth.tmc.edu; 2 Translational Genomics Research Institute, (TGen), Phoenix, AZ 85004, USA; dvh@tgen.org; 3 Department of Molecular Diagnostics and Experimental Therapeutics, Beckman Research Institute, City of Hope, CA 91010, USA; ajgoel@coh.org

**Keywords:** pancreatic cancer, exosomes, extracellular vesicles, early detection, biomarkers

## Abstract

Pancreatic cancer remains one of the deadliest diagnoses a patient can receive. One of the reasons for this lethality is that this malignancy is often detected very late due to a lack of symptoms during the early stages. In addition to the lack of symptoms, we currently do not have a reliable biomarker for screening. Carbohydrate antigen (CA) 19-9 has a sensitivity between 79% and 84% and a specificity of 82–90%, making it unreliable for early detection. Recently, there have been numerous studies on the use of extracellular vesicles (EVs) to detect pancreas cancer. This field has been rapidly expanding, with new methods and biomarkers being introduced regularly. This review provides a systematic update on the commonly used and promising methods used in the detection of EVs, biomarkers associated with EVs for early detection and prognosis, as well as studies looking at using EVs as therapeutics. The review ends with remarks about areas to focus on using EVs going forward.

## 1. Introduction

Pancreatic cancer (PC) is one of the leading causes of cancer-related deaths worldwide [1]. Unfortunately, due to the lack of widely accepted tools for the early detection of PC and a lack of apparent symptoms during early stages, PC is often detected at later stages of the disease. The late detection of PC leads to a dismal worldwide five-year survival rate of less than 9% [2]. Carbohydrate antigen (CA) 19-9, the current primary biomarker for PC, has a sensitivity between 79% and 84% and a specificity of 82–90% [3,4]. However, it has limited utility in early detection due to false positives in patients with other diseases, such as chronic pancreatitis, cholangitis, and other malignancies [5].

Recently, many studies have highlighted the potential clinical significance of interrogating EVs as possible biomarkers for the early detection of PC [5]. There are many different types of EVs. The most commonly referred to in the literature are exosomes, which are approximately 30–150 nm in size [6]. In the present review, we consistently refer to the broader population termed EVs. These vesicles are secreted by almost all cell types and can be found in many different fluids, including plasma, saliva, urine, and human milk [7]. The ubiquity of EVs and the noninvasive methods that can be used to collect them make them excellent potential biomarkers [8,9]. One major limitation to developing EVs as biomarkers has been the difficulty and expense associated with isolating, quantifying, and characterizing them. However, recent developments have made these tests more practical. 

**Figure 1:**
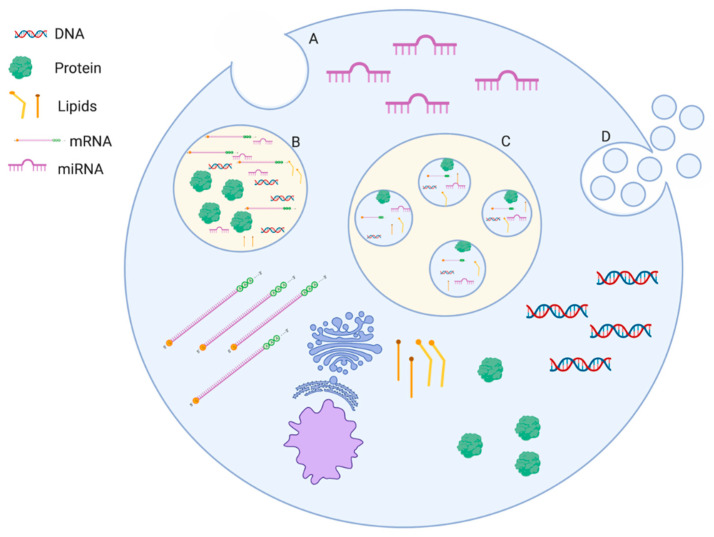
Biogenesis of Exosomes. Label A illustrates endocytosis of the plasma membrane. Label B depicts the uptake of different materials found in the cytosol. Label C shows the formation of multivesicular bodies. Ending with the eventual release of the exosomes through exocytosis shown by label D. It is important to note that a recent study by Jeppesen et al. called into question the presence of DNA inside of exosomes.

The biogenesis of exosomes has been outlined in great detail in many reviews [8,9,10,11]. Briefly, exosome biogenesis starts with the formation of early endosomes through internal budding of the lipid bilayer of the cell, labeled as “A” in Figure 1 below. 

As the early endosome develops into the late endosome, it incorporates various intracellular molecules, including mRNA, miRNA, DNA, proteins, and lipids labeled as “B” in Figure 1. Exosome biogenesis continues with internal budding of the lipid bilayer of a late endosome or multivesicular body (MVB), (depending on nomenclature used) labeled as “C” in Figure 1. The MVB is packaged with mRNA, miRNA, DNA, proteins, and lipids that were taken up by the endosome earlier. It is important to note that a recent study calls into question whether or not DNA is present in exosomes. In that study, small EVs with traditional exosomal markers were separated from the extracellular compartment using high resolution density gradient fractionation and direct immunoaffinity capture. When characterized, it was shown that the active DNA that was previously presumed to be associated with EVs was absent. The presence of DNA observed in other approaches may be due to insufficient purification that is resolved via improved methodology [12]. An extensive database can be found at http://www.exocarta.org/ for proteins, RNA, and lipids that have been found in exosomes. Exosomes are then released into the extracellular space through exocytosis of the MVB labeled as “D” in Figure 1. The material they are packaged with indicates they likely have a role in cell-to-cell communication.

**Figure 2:**
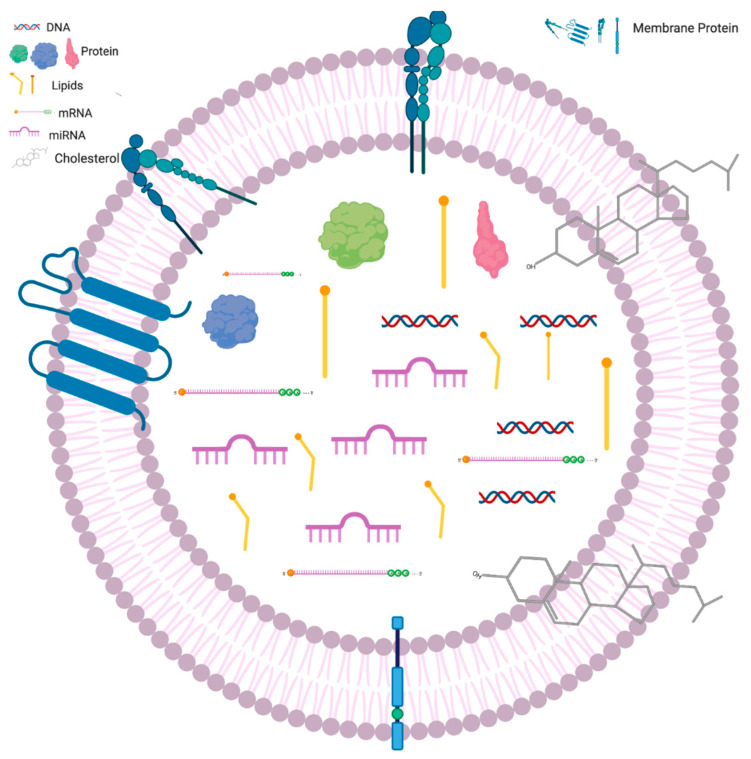
Composition of EVs. Exosomes comprise a heterogeneous lipid bilayer with a variety of biological molecules such as integrins and cholesterol. Within are lipids, proteins, DNA sequences, mRNA, and miRNA. Recent results from Jeppesen et al. called into question the presence of DNA inside of exosomes.

Upon release from the cell, the exosome consists of a heterogeneous lipid bilayer interspersed with various membrane proteins and cholesterol [13]. Pang et al. produced excellent images using electron microscopy that can be found in their paper on dual surface enhanced Raman spectroscopy (SERS). The internal composition of an exosome consists of a variety of proteins, mRNA, lipids, and possibly DNA. However, as mentioned previously, that has been called into question recently [12]. The external and internal composition of EVs is outlined in Figure 2 below.

It is the composition of EVs, specifically exosomes, that make them a potential biomarker for cancer detection. The internal and external composition of EVs change based on the cell line from which they originate. This specificity, coupled with the fact that, in the setting of cancer, EV production is increased, constitutes the basis of the hypothesis of using them as biomarkers for early detection of PC [14].

**Figure 3:**
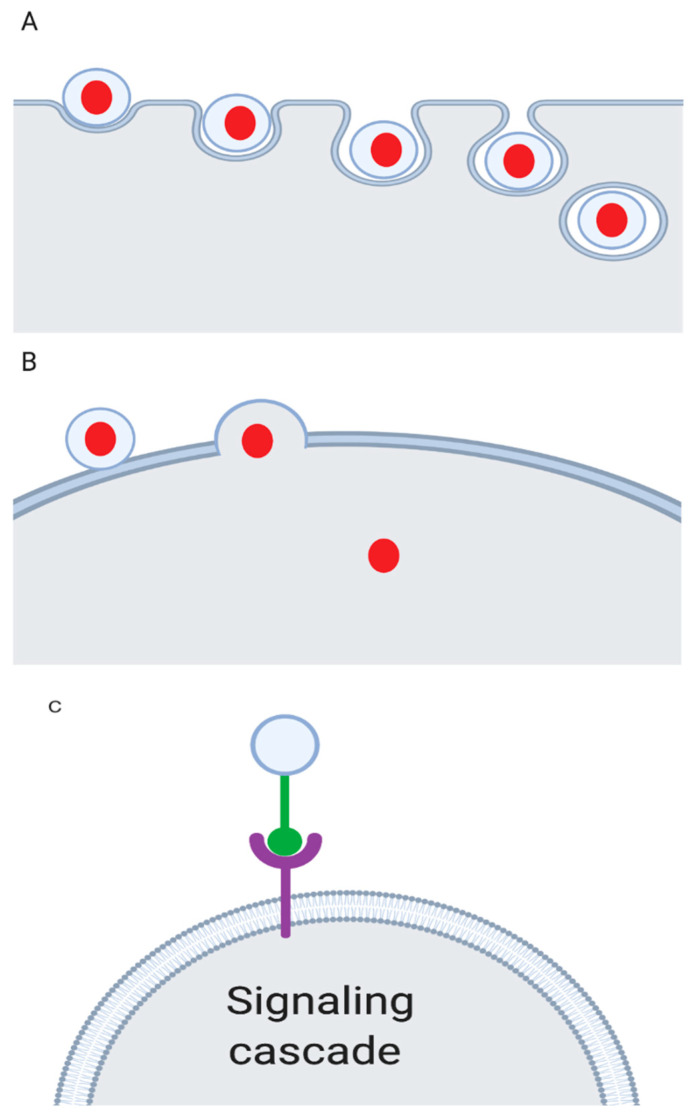
Proposed EV signaling mechanisms. Panel (**A**) depicts endocytosis of the EV into a cell. Panel (**B**) depicts the fusion of an EV with the cell membrane then depositing material in the cell. Panel (**C**) shows a receptor-ligand interaction leading to a downstream signaling cascade.

How EVs participate in cell-to-cell communication is still under investigation. One hypothesis is that EVs signal through surface proteins found on their membranes. The proposed mechanism is synergistic signaling through interaction between membrane proteins on the EV with membrane proteins on cells [13]. Because the ability to identify the origin of EVs is still developing, it remains challenging to identify whether they are targeted or are released systemically and communicate more broadly without specific cellular tropisms. EVs appear to have multiple mechanisms interacting with recipient cells. They can fuse with the cell membrane of their target cell as well as be endocytosed, or engage with cell surface proteins and thereby initiate a signaling cascade [13]. Figure 3 illustrates the proposed EV communication mechanisms, including fusion with the target cell membrane, endocytosis by the target cell, and the binding with a cell surface membrane triggering a signaling cascade in panels A, B, and C, respectively.

In addition to being early biomarkers, EVs have been explored as prognostic factors [15]. Investigators have also started exploring the utility of EVs in therapeutics. Using EVs in therapeutics has been studied both as direct targets by preventing their release or indirect targets by attempting to interfere with the signaling mechanism for intercellular communication. EVs are also being studied as drug delivery vehicles to overcome the inherent chemoresistance of pancreatic fibroblasts [10,16].

This review will provide a brief overview of some commonly used lab techniques for detection, isolation, and characterization of EVs, using EVs as biomarkers for early detection of PC, their use in the prognosis and monitoring of PC, and the use of EVs in therapeutics for PC. It will close with thoughts regarding next steps for EVs relating to PC. 

## 2. Methods

The PubMed database was searched using the words: pancreas, pancreatic, cancer, EVs, and exosomes in different combinations. The abstracts of the search results were reviewed. If the abstract was deemed relevant, then the study was reviewed in earnest and summarized as appropriate, then used and cited in this review.

The figures were designed using the BioRender website at biorender.com [17].

## 3. Isolation and Analysis Techniques

An overview of all the techniques is provided in Table 1 below.

### 3.1. Isolation Techniques

#### 3.1.1. Differential Centrifugation Coupled with Ultracentrifugation

Differential centrifugation is the process of spinning down a sample and fractionating it by the size and density of particles, leaving a supernatant and a pellet at the top and bottom of the sample, respectively. When coupled with ultracentrifugation, the process allows the investigator to screen for specific particles in a sample [20].

Advantages associated with this are resultant sample purity, low additive contamination, and low cost. These advantages come from the specificity of the pellet, the minimal need for additives, and the minimal equipment and product requirements. The disadvantages of this method are low output, time required, potential damage, and contamination of protein aggregates [18,22,37,38]. These disadvantages come from the nature of ultracentrifugation given that minimal numbers of samples can be tested at a time, the high speeds at which samples have to be spun, and potential for contamination from proteins found in the sample. Ultracentrifugation has been labeled as the gold standard by nature of being the first technique used to isolate and characterize EVs, and it remains the most commonly used method for isolation [19,25,39]. A review by Witwer et al. in 2013 points out the downsides of ultracentrifugation, such as how time-consuming it is and the potential for contamination. In 2015, Zarovni et al. mentioned that ultracentrifugation was the most commonly used technique [40]. However, they called into question if it should be considered the gold standard when presenting their use of immunoisolation techniques.

#### 3.1.2. Differential Centrifugation Coupled with Ultracentrifugation Plus Density Gradient

When adding a density gradient to ultracentrifugation, the primary purpose is to purify further small molecules such as proteins and enzymes [20]. Typically, this is done using a sucrose gradient that allows for further separation of EVs [22].

In a review done by Konoshenko et al. in 2018, it was found the ultracentrifugation with some sort of modification was the most commonly used method in the studies. Ultracentrifugation with a density gradient was used in 11.6% of the studies reviewed [41]. Adding a density gradient with centrifugation is the most effective method with the highest purity used for detecting EVs in blood samples. The advantages of this technique are the resultant purity, low contamination from materials used, best value for protein, and preservation of mRNAs and miRNAs. These advantages are similar to those found in general ultracentrifugation with the added purity from adding the density gradient. The disadvantages are low output, labor and equipment intensive, contamination with high density lipoprotein, and potential damage [18,22,37,38].

#### 3.1.3. Polymer-Based Precipitation

This technique involves using a polymer to precipitate EVs out of a supernatant. The vesicles are precipitated out with a polymer, such as polyethylene glycol, washed, then ultracentrifuged again to remove any additional molecules that might have been coprecipitated by the initial introduction of the polymer [23].

This technique is useful for detecting EVs in cell supernatant but not in blood samples. Its advantages are ease of performance, high output, and no special equipment needed. However, if profiling of specific EVs is the primary goal, this is an inappropriate technique. There is contamination with both polymer and lipoprotein, and any RNA present is lost in the isolate. In addition, it is time-consuming [18,23].

#### 3.1.4. Immune Capture Isolation

In this technique, EVs are isolated based on the known proteins and receptors found on their membranes [25]. After this technique is performed, the isolated sample is placed through ultracentrifugation to isolate further EVs [24].

This method works well with cell supernatant and is specific for selected EV subtypes. The disadvantages are antibody and non-vesicular protein contamination because they are required to isolate the EVs, time-consuming, as well as low output [18].

#### 3.1.5. Size Exclusion Filtration

This method filters particles by molecular weight. Typically, this is performed using a gel membrane column to filter the desired products and is commonly used as the final step in a purification process [26].

The advantages of this technique include high output, fast procedure, and resultant purity. Disadvantages include the poor quality of protein, mRNA, miRNA, and the deformation and destruction of EVs [18].

### 3.2. Analysis of Vesicular Materials

#### 3.2.1. Surface Enhanced Raman Spectroscopy (SERS) Biosensor

In short, SERS relies on two mechanisms for the detection of EVs. One mechanism is the excitation of surface plasmons leading to the amplification of light, registering a detectable signal. The amplification of light usually occurs in gaps, crevices, or sharp borders of plasmonic materials. The second mechanism SERS uses to operate is chemical enhancement, where the excitation wavelength is resonant with the metal being used, which leads to a charge transfer [30].

SERS appears to be a promising potential method for the detection of miRNA in EVs [42,43]. It has the benefit of being fast and inexpensive to perform [28]. Of note, Pang et al. used a duplex-specific nuclease (DSN) assisted dual-SERS biosensor for a one-step approach to quantify microRNA. Also, SERS has been shown to enable single vesicle identification, thereby showing the potential to gather information about ratios of specific vesicles in a mixture [43]. In another proof of concept, SERS was coupled with principal component differential function analysis (PC-DFA). The authors found that when SERS was coupled with PC-DFA, the technique had a sensitivity and specificity of 90.6% and 97.1%, respectively, and distinguished pancreatic cell lines from a healthy cell line [42]. The drawbacks associated with SERS include that it has weak signals when working with biological samples and the signals can be challenging to interpret [27,29,44]. Weak signals in biological samples can be overcome by using surface enhancement [29]. SERS is an active area of current research, and new technologies are being created regularly, as evidenced by Tian et al. in 2018 [5].

#### 3.2.2. Nanoplasmon Enhanced Scattering (nPES) Assay

nPES is another new and promising method for the identification, quantification, and characterization of EVs. This technique works through gold spheres (AuS) and gold rods (AuR) scattering green and red light, respectively. The spheres and rods are targeted to bind molecules specific to vesicular membranes, such as CD81, which form complexes, leading them to scatter light, creating a yellow glow. The intensity of the glow corresponds to the strength of the signal. nPES allows for both detection and quantification of EVs in a sample. Also, targeting either the gold rods or spheres to specific biomarkers allows for the characterization of the EVs [9,31].

The main advantage of nPES is that it can be used with a sample as small as one microliter of plasma to identify EVs [31]. Liang et al. also found that they did not need to purify their sample, taking costly and time-consuming steps out of the process. The drawbacks associated with this technique include it, as a newer technology, not being widely available, the weak signal associated with biological samples, and it currently requires specialized equipment [32,43].

#### 3.2.3. Digital PCR

This technique uses four steps: droplet generation, PCR amplification, droplet reading, and data analysis. During droplet generation, the machine takes the sample and generates droplets that contain the target sequence of nucleic acids. PCR amplification replicates the target nucleic acid sequence for easier identification. The reader identifies samples with the target sequence, which is then analyzed during data analysis [33].

The primary benefit of this method is that it can be used to detect vesicular DNA. Yang and colleagues used digital PCR to identify the presence of mutant KRAS and mutant TP53 genes in EVs in patients with PC. The investigators set out to identify the frequency at which digital PCR could identify the presence of the mutation. The threshold for the detection was set at 0.25% of spiked mutations for digital PCR to detect the KRAS and TP53 mutations using vesicular DNA reliably [37,38]. The samples were evaluated by measuring the relative functional abundance in different titrated samples of mixed samples of vesicular DNA that contained both mutated and wild types of the genes to find the appropriate cut-off for a positive test [34]. However, this is not a reasonable method for detection at low concentrations of EVs [34]. Additionally, it cannot detect other molecules found in EVs such as proteins and lipids, which are critical for classifying EVs.

#### 3.2.4. Mass Spectroscopy for Proteomics

On a basic level, mass spectroscopy works through converting molecules into gas-phase ions, which are then measured, providing a mass-to-charge ratio. To analyze proteins, electrospray ionization, or matrix-assisted laser desorption/ionization are added to the mass spectrometer. These are soft ionization techniques that allow for the identification of proteins [35,36].

The main advantage of this method is the ability to detect proteins, both within EVs and associated with the lipid bilayer. It is a noncomprehensive technique in the evaluation of EV characteristics due to its inability to detect genetic material such as miRNA [36].

## 4. Cancer Detection/Prognosis/Monitoring

Table 2 below summarizes the vesicular biomarkers that have been investigated for the detection, prognosis, and monitoring of PC.

### 4.1. Detection

Glypican-1 (GPC-1) is one of the most studied biomarkers for PC found on EVs to date. In 2015, Melo et al., using ultracentrifugation to isolate EVs from both animal and human cell lines, reported a 100% sensitivity and specificity when using transmission electron microscopy detection of GPC-1 on EVs for stage 1 PC. When using ELISA to detect the presence of GPC-1, the sensitivity and specificity decreased to 82.14% and 75%, respectively, with a positive predictive value of 4% and a negative predictive value of 100% [63]. Buscail et al., using a Total Exosome Isolation kit from Thermofisher involving ultracentrifugation and an isolation/detection reagent to pull down sera exosomes, attempted to replicate these findings by looking at using GPC-1 as a diagnostic marker and compared the results to using CA 19-9 alone, endoscopic ultrasound with fine needle biopsy (EUS FNA) alone, as well as all three combined [64]. They found that using EVs from peripheral or portal vein samples to have a sensitivity of 64% and specificity of 90%, which was more sensitive than EUS FNA and/or CA19-9. When all three modalities were combined, they had a sensitivity of 82% and a specificity of 86%. The best diagnostic accuracy, 84%, was obtained when using all three combined or combining just GPC-1 EVs with CA19-9.

An earlier attempt to replicate the GPC-1 results by Melo et al. using a more clinically viable detection method showed that when using ELISA to detect GPC-1-enriched EVs, there was no significant difference between patients with pancreatic adenocarcinoma (PDAC) and patients with benign pancreatic conditions. The EV isolation technique used in this study was ultracentrifugation [65]. At the end of their study, Frampton et al. called for a standardization of the technique to detect circulating tumor EVs. Thereby making the findings more consistent, easier to compare from study to study, and allow for better replication studies.

One struggle in finding a reliable biomarker is the ability to distinguish early stage disease from other benign pancreatic processes. EphA2 has been explored as a possible vesicular biomarker. Liang et al. used ultracentrifugation to isolate EVs from cell media and then again to prepare them for ELISA using ExoQuick kit from SBI; they reported a sensitivity and specificity of 91% and 85%, respectively, when distinguishing stage I and II PC from healthy controls. In addition, they reported a sensitivity and specificity of 86% and 85%, respectively, when distinguishing stage I and II disease from pancreatitis [31].

Another marker that has been investigated for early detection of PC is EVs containing c-Met. In this study, ultracentrifugation was combined with Invitrogen Total Exosome Isolation Reagent to isolate the EVs [66]. However, the sensitivity and specificity were not statistically different from using CA19-9 to detect PC.

Zou et al., in 2019, investigated the potential of miRNA in EVs to aid in the early detection of PC [67]. They found that EVs isolated from PC patient serum samples, using ExoQuick Exosome Precipitation Solution followed by centrifugation to isolate the exosomes, had significantly higher levels of miR-192-5p, miR-19a-3p, and miR-19b-3p when compared to controls. Pang et al., in 2019, in their dual SERS experiment showed significantly higher levels of miR-10b in the setting of PC than either normal controls or patients with chronic pancreatitis.

It was found that both miR-17-5P and miR-21 were both significantly elevated in PDAC patients. These results were obtained by isolating EVs using a 0.22-μm filter followed by ultracentrifugation, then EVs were collected using a *mir*Vana PARIS RNA isolation kit (Ambion, Austin, Tx, USA) [68]. miR-21 is also found to be elevated in multiple solid tumors, raising the possibility that it is a common mechanism in carcinogenesis for multiple cancers. The presence of elevated levels of miR-21 in multiple solid tumors could also limit its use in early detection of PC due to a lack of specificity [57].

Yang et al., in 2017, used multiple biomarkers known to be associated with PDAC in an assay. They created a panel they called PDAC^EV^ that consisted of EGFR, EpCAM, HER2, MUC1, GPC-1, and WNT2. Yang et al. used media that was filtered through a 0.22 µm cellulose acetate vacuum filter (Corning, 430767) followed by two rounds of ultracentrifugation split by washing with PBS. When analyzed, the training cohort (n = 6) had a sensitivity of 100%, a specificity of 100%, and an accuracy of 100%. When analyzed, the prospective cohort (n = 43) had a sensitivity of 95%, a specificity of 81%, and an accuracy of 88%. The use of a panel of multiple markers is a practical avenue to continue to explore, given the lack of replicable studies using just one biomarker.

### 4.2. Monitoring and Prognosis of PC

It has been shown that increased levels of *ANXA6^+^*correlate with a worse prognosis. There is also some evidence showing that the levels can be used to predict how advanced the cancer is at diagnosis. To obtain these findings, the investigators used Total Exosome Isolation Reagent (Invitrogen) according to the manufacturer’s instructions to isolate the EVs [48]. Another vesicular biomarker that has been proposed as a prognostic indicator is programmed death-ligand 1 (PD-L1). Using ultracentrifugation combined with Invitrogen Total Exosome Isolation Reagent to isolate the EVs, patients with a high PD-L1 burden detected on circulating EVs had a statistically significant shorter average post-surgical resection survival times, 7.8 months versus 17.2 months [66]. Zhuan-Sun et al. did a meta-analysis that showed higher levels of PD-L1 in tissue samples was a negative predictor for the overall survival of PDAC patients [69].

Vesicular epithelial cell adhesion molecule (EpCAM) has also been investigated as a potential biomarker for prognosis. The investigators isolated the EVs with two rounds of ultracentrifugation. Patients with higher levels of EpCAM before initiating treatment in the setting of either metastatic or nonresectable locally advanced PDAC had shorter progression free and overall survival times. Interestingly, the same study also showed that an increase in levels of EpCAM present during treatment was associated with a better overall prognosis [70].

One prognostic factor in all cancers is the presence of metastases. It has been demonstrated that EVs derived from PDAC create a fibrotic environment for the liver, priming it for metastases as a result of a multistep mechanism [15]. PC-derived EVs induce the activation of profibrogenic activities, including proliferation, migration, collagen production, and alpha-SMA mRNA expression, which creates pro-metastatic niches [71]. Even in early stage I disease in mice, the presence of EVs with macrophage migratory inhibitory factor prepped the liver for metastases. This was done by increasing fibronectin deposition, tumor growth factor beta signaling, and recruitment of bone-marrow macrophages. When these effects were targeted by treatment, the occurrence of liver metastases decreased [72].

The amount of GPC-1 has also been shown to have a positive correlation with distant metastatic disease [63]. In the replication study that Buscail et al. published in 2019, they showed patients with GPC-1 positive EVs detected in the peripheral blood had a worse prognosis. Interestingly, the same relationship was not seen when GPC-1 positive EVs were detected in the portal system. When Frampton et al. attempted to replicate the GPC-1 study by Melo et al., they found that the degree of elevation of GPC-1 EVs was correlated with the tumor burden as well.

Tumor associated EVs have also been shown to carry and deposit anti-apoptotic proteins and mRNAs, including Survivin, cIAP1, cIAP2, and XIAP, implicating them in the progression and expansion of tumors. These results were obtained by isolating the EVs through ultracentrifugation, followed by treatment with ExoQuick TC™. After incubation with ExoQuick TC™, the samples were resuspended preparing them for further analysis [55].

In addition to being a potential tool for the early detection of PC, miR-21 was found to be associated with metastatic and advanced disease [68]. One possible explanation is that miR-21 is associated with matrix metalloproteinases 2 and 9 as well as VEGF, which all promote migration and tumor invasion [57].

## 5. Therapeutics

Exploring the use of EVs in therapeutics is still in the early stages. Batista and Melo wrote an excellent review in 2019 on the methods that had been explored to date. They noted two different mechanisms that have been explored. One such difference is using EVs as a delivery vehicle for systemic treatment, while the other is by blocking either the biogenesis or release of EVs, thereby preventing cancer promoting signaling from the EVs. Both will be examined in turn.

### 5.1. EVs as Delivery Vehicles

Kamerkar et al. in 2017 performed a study with EVs derived from normal fibroblast-like mesenchymal cells that were engineered with siRNA or shRNA targeting oncogenic KRAS^G12D^ (iExosomes). They analyzed mice with a KRAS^G12D^ mutation and showed a reduction in tumor size after 30 days of treatment compared to control mice not given iExosomes. When compared to liposomes loaded with the same siRNA or shRNA, the reduction in size was greater in the iExosome group. This finding persisted after 200 days of treatment with iExosomes. The proposed mechanism of these findings was suppressed signaling from KRAS and suppressed KRAS^G12D^ expression in tumors and pancreas. The result was improved survival in the mice receiving iExosomes. Using these EVs did not seem to impact KRAS^WT^ [73].

Recently, efforts have been made towards engineering cells that produce custom therapeutic exosomes with a specific surface protein composition, such as IL-12, to target T- and NK-cells. Alternatively, engineering exosomes with a specific glycoprotein to facilitate a selective cellular tropism, combined with exogenous loading of a potent therapeutic agent, such as a cyclic dinucleotide, to stimulate the STING pathway in mature antigen presenting cells has also been explored as a potential therapeutic mechanism [74,75,76].

### 5.2. Disrupting Production and Signaling of EVs

Chemoresistance is one of the critical factors in the struggle to treat PC effectively. One of the most commonly used drugs currently is gemcitabine (GEM). Unfortunately, after treatment with GEM, the EVs released typically imbue the area with increased resistance to GEM [16]. Richards et al. in 2017 showed that treatment with GEM led to a more than seven-fold increase in the release of EVs from cancer associated fibroblasts (CAF). Increased release of EVs resulted in a marked increase in miR-146a and mRNA for Snail, both of which are known to promote chemoresistance, epithelial-to-mesenchymal transition, and metastasis in colorectal cancer [77]. Richards et al. took the next step and blocked the release of EVs containing miR-146a from CAF using the drug GW4869. They observed that multiple cell lines maintained chemosensitivity.

When incubated with GEM, PC cells, via EVs, upregulate the expression of miR-155 and transfer miR-155 to other PC cells leading to suppression of apoptotic pathways [19]. PC-derived EVs have been shown to suppress the immune system by downregulating HLA-DR expression, thus reducing antigen display [19]. Taking PC-derived EVs and depleting them of miRNAs has been shown to increase the tumor-killing capacity of dendritic cells as well as other anti-tumor cells in the body [19].

## 6. Going Forward

### 6.1. Detection Methods

Both nPES and SERS biosensors show promise as cost-effective and rapid methods for detecting and characterizing EVs from serum or plasma samples. Both methods should continue to be improved and evaluated for their strengths and weaknesses, including the need for purification, the amount of sample needed, and the cost that it would transfer to the patient. One specific area for improvement should be focusing on the quantification of biomarkers of interest [78].

### 6.2. Early Detection

Using EVs as biomarkers for early detection should continue to be investigated. However, a highly sensitive and specific test using a single biomarker seems unrealistic. Based on the study performed by Yang et al. in 2017, an assay using multiple biomarkers on EVs is a promising area for further investigation [79].

Trials in high risk patient populations make the most sense at this time. These trials will provide opportunities to evaluate the utility of EVs for detecting PC in the early stages and distinguish it from other benign pancreatic conditions. Since PC is regularly not detected until late in the disease process, these early detection tests could be instituted with great potential benefit.

### 6.3. Novel Therapeutic Modalities

Given the role EVs play in intercellular signaling, they offer a unique and novel mechanism for the delivery of therapy [80]. Targeting EVs to specific cell types should be explored, potentially allowing us to reach immune privileged sites and reduce treatment-related toxicities.

EVs could also be the target of the treatment by preventing release or interrupting their signaling to other cells. However, our understanding of EVs’ role in cell-to-cell communication remains incomplete. It is possible that interfering with EV signaling could lead to toxicities unacceptable to patients. Hoshino et al. in 2015 showed that specific integrins on tumor-derived EVs played a role in preparing a niche for metastases to specific organs [81]. If it is possible to target the site of action on the organ where the EVs act or the EV itself, this could theoretically increase progression free survival times.

## 7. Conclusions

Overall, EVs offer promise as biomarkers for the early detection and monitoring of PC. Investigations should continue until EV-based approaches reach adequate sensitivity and specificity to be considered a reliable detection test. Using EVs in therapeutics should also continue to be investigated as it offers the potential to provide an effective treatment for PC. To date, PC is a disease that is generally detected very late and is challenging to cure. EVs have the potential to provide tools to detect PC early in the disease process with very high specificity and sensitivity and to treat it.

## Figures and Tables

**Table 1:** Summary of isolation and analysis of new and commonly used and new techniques for EVs.

Technique	Use	Advantages	Drawbacks	References
**Differential centrifugation coupled with ultracentrifugation**	Isolation	High purity Low additive contamination Low cost	Low output Time-consuming Otential damage Contamination of protein aggregates	[18,19,20]
**Differential centrifugation coupled with ultracentrifugation plus density gradient**	Isolation	High purity Low contamination Best value for protein Preserves mRNAs and miRNAs.	Low output Labor and equipment intensive Contamination with high density lipoprotein Potential damage	[18,20,21,22]
**Polymer-based precipitation**	Isolation	Easy to perform High output Fast No special equipment needed	Low purity Polymer contamination Lipoprotein contamination Poor RNA preservation	[18,23]
**Immune capture isolation**	Isolation	Efficacious with supernatant High specificity for selected EV subtypes	Antibody contamination Non-vesicular protein contamination Low output Time-consuming	[18,24,25]
**Size exclusion filtration**	Isolation	High output Relatively quick Reasonable purity	Poor quality of protein Poor quality of mRNA Poor quality of miRNA, Deformation of EVs Destruction of EVs	[18,26]
**Surface enhanced Raman spectroscopy (SERS) Biosensor**	Identification, quantification, and characterization	One step process	Weak biological signals Difficult interpretation	[5,27,28,29,30]
**Nanoplasmon enhanced scattering (nPES) assay**	Identification, quantification, and characterization	One step process Can detect multiple biomarkers	Not widely available Specialized equipment required Weak signal with biological samples	[29,31,32]
**Digital PCR**	Analysis	Can detect DNA and RNA	Requires a high concentration of EVs No protein detection	[33,34]
**Mass Spectrometry for Proteomics**	Analysis	Detects both internal and external components	No detection of nucleic acids	[35,36]

**Table 2:** Summary of extracellular vesicle biomarkers used in PC.

Marker	Molecule Type	Detection	Prognosis	Monitoring	Proposed Function in Pancreas Cancer
**Glypican-1**	Protein	Yes	Yes	Yes	Cell division Growth regulation [19]
**EphA2**	Protein	Yes	Yes	Yes	Cell Migration [45]
**EpCAM**	Protein	Yes	Yes	Yes	Cell-cell adhesion [46] Proliferation [46] Maintenance of undifferentiated states [46] Regulation of differentiation [46]
**c-Met**	Protein	Yes	Unknown	Unknown	Proliferation [47] Motility [47] Migration [47] Invasion [47]
**ANXA6+**	Protein	Yes	Yes	Yes	Survival Migration [48]
**PD-L1**	Protein	No	Yes	Unknown	Immune evasion [49]
**Macrophage Migration Inhibitory Factor (MIF)**	Protein	No	Yes	Unknown	Immune evasion [50]
**HER2**	Protein	Yes	Unknown	Unknown	Carcinogenesis [51]
**MUC1**	Protein	Yes	Yes	Unknown	Chemoresistance Metastases [52]
**WNT2**	Protein	Yes	Unknown	Unknown	Metastases [53] Cell cycle progression [53] Anti-apoptotic [53] Chemoresistance [53]
**EGFR**	Protein	Yes	Yes	Unknown	Carcinogenesis [54]
**Survivin**	Protein	Unknown	Unknown	Unknown	Anti-apoptotic [55]
**cIAP1**	Protein	Unknown	Unknown	Unknown	Anti-apoptotic [55]
**cIAP2**	Protein	Unknown	Unknown	Unknown	Anti-apoptotic [55]
**XIAP**	Protein	Unknown	Unknown	Unknown	Anti-apoptotic [55]
**miR-17-5p**	microRNA	Yes	No	Unknown	Tumor Suppressor [56]
**miR-21**	microRNA	Yes	Yes	Unknown	Migration [57] Tumor invasion [57]
**miR-192-5p**	microRNA	Yes	Unknown	Unknown	Unknown
**miR-19a3p**	microRNA	Yes	Unknown	Unknown	Cell Proliferation [58] Anti-apoptotic [58]
**miR-19b-3p**	microRNA	Yes	Unknown	Unknown	Cell Proliferation [59]
**miR-10b**	microRNA	Yes	Unknown	Unknown	Tumor Invasion [60]
**miR-155**	microRNA	No	Yes	Unknown	Chemoresistance [21] Tumor invasion [61] Migration [61]
**miR-203**	microRNA	Unknown	Unknown	Unknown	Immune evasion. [62]

## Abbreviations

**Definitions**	
Surface Plasmon	delocalized electron that oscillates between any two materials
**Abbreviations**	
CA 19-9	Carbohydrate Antigen 19-9
CAF	Cancer associated fibroblast
DSN	Duplex-Specific Nuclease
EUS FNA	Endoscopic Ultrasound with fine needle biopsy
EV	Extracellular Vesicle
GEM	Gemcitabine
GPC1	Glypican-1
nPES	Nanoplasmon enhanced scattering
PC	Pancreatic Cancer
PC-DFA	Principal Component Differential Function Analysis
PDAC	Pancreatic adenocarcinoma
PD-L1	Programmed Death-Ligand 1
SERS	Surface enhanced Raman spectroscopy
